# Long-Term Outcomes of Internet-Based Self-Management Support in Adults With Asthma: Randomized Controlled Trial

**DOI:** 10.2196/jmir.2640

**Published:** 2013-09-12

**Authors:** Johanna L van Gaalen, Thijs Beerthuizen, Victor van der Meer, Patricia van Reisen, Geertje W Redelijkheid, Jiska B Snoeck-Stroband, Jacob K Sont, SMASHING Study Group

**Affiliations:** ^1^Leiden University Medical CenterDepartment of Medical Decision MakingLeidenNetherlands

**Keywords:** asthma, quality of life, self-management, long-term, eHealth, Internet, telemedicine

## Abstract

**Background:**

Long-term asthma management falls short of the goals set by international guidelines. The Internet is proposed as an attractive medium to support guided self-management in asthma. Recently, in a multicenter, pragmatic randomized controlled parallel trial with a follow-up period of 1 year, patients were allocated Internet-based self-management (IBSM) support (Internet group [IG]) or usual care (UC) alone. IBSM support was automatically terminated after 12 months of follow-up. In this study, IBSM support has been demonstrated to improve asthma-related quality of life, asthma control, lung function, and the number of symptom-free days as compared to UC. IBSM support was based on known key components for effective self-management and included weekly asthma control monitoring and treatment advice, online and group education, and communication (both online and offline) with a respiratory nurse.

**Objective:**

The objective of the study was to assess the long-term effects of providing patients 1 year of IBSM support as compared to UC alone.

**Methods:**

Two hundred adults with physician-diagnosed asthma (3 or more months of inhaled corticosteroids prescribed in the past year) from 37 general practices and 1 academic outpatient department who previously participated were invited by letter for additional follow-up at 1.5 years after finishing the study. The Asthma Control Questionnaire (ACQ) and the Asthma Quality of Life Questionnaire (AQLQ) were completed by 107 participants (60 UC participants and 47 IG participants). A minimal clinical important difference in both questionnaires is 0.5 on a 7-point scale.

**Results:**

At 30 months after baseline, a sustained and significant difference in terms of asthma-related quality of life of 0.29 (95% CI 0.01-0.57) and asthma control of -0.33 (95% CI -0.61 to -0.05) was found in favor of the IBSM group. No such differences were found for inhaled corticosteroid dosage or for lung function, measured as forced expiratory volume in 1 second.

**Conclusions:**

Improvements in asthma-related quality of life and asthma control were sustained in patients who received IBSM support for 1 year, even up to 1.5 years after terminating support. Future research should be focused on implementation of IBSM on a wider scale within routine asthma care.

**Trial Registration:**

International Standard Randomized Controlled Trial Number (ISRCTN): 79864465; http://www.controlled-trials.com/ISRCTN79864465 (Archived by WebCite at http://www.webcitation.org/6J4VHhPk4).

## Introduction

Asthma is a common chronic disease with a prevalence of approximately 6% among adults [1]. It is characterized by chronic inflammation and/or structural changes of the airways, which leads to recurrent episodes of wheezing, coughing, difficulty breathing, and/or chest tightness [2,3]. According to clinical guidelines [2,3], treatment strategies for asthma should be aimed at minimization of symptoms, optimization of lung function, and prevention of symptom aggravation with few medication side effects. Even though effective therapies are widely available, many patients do not achieve these treatment goals [1,4]. As a consequence, asthma still imposes a significant burden of disease on the individual patient. A proactive patient-centered approach consisting of education, treatment goals, self-monitoring, and an action plan, accompanied by guidance and regular review by a health care provider, has the potential to improve outcomes in asthma [5], including improved quality of life and a reduced number of hospitalizations and unscheduled doctor visits. In spite of the prominent role within guidelines, adoption of this “guided self-management” is lacking [6]. While many practices do offer patients a routine medical review, only a minority of patients are provided with an action plan by their health care provider [7,8]. Usage of action plans by patients could be enhanced if action plans are part of a patient-professional partnership and when they are tailored to the needs of the individual patient [9].

Provision of Internet technology has been proposed as an appealing medium for asthma management [10-14]. Indeed, in the study by Van der Meer et al [15] in patients with mild to moderate persistent asthma, it was demonstrated that provision of an Internet-based self-management (IBSM) support program for 1 year leads to improved asthma-related quality of life, asthma control, lung function, and the number of symptom-free days as compared to usual care (UC) alone. A post hoc analysis of this study [16] demonstrated that patients with asthma that was not well controlled benefited the most from IBSM support. In addition, this study showed that at 12 months of follow-up, about 60% of the patients were still using the program of their own initiative. However, it is unknown whether the benefits are sustained over a long-term period. We hypothesized that the benefits of providing 1 year of IBSM support are sustained over a long-term period.

In this paper, we aim to assess the long-term effects of providing patients 1 year of IBSM support as compared to UC alone.

## Methods

### Participants

Two hundred patients who previously participated in a 12-month multicenter, nonblinded, pragmatic randomized controlled parallel trial were invited for additional follow-up 1.5 years after finishing the study. Full details of the study methodology and subjects for the Self-Management of Asthma Supported by Hospitals, ICT, Nurses and General practitioners (SMASHING) study have been published elsewhere [15]. Briefly, patients were recruited from 37 general practices in the Leiden and The Hague area and from the outpatient clinic of the department of Respiratory Medicine of the Leiden University Medical Center (LUMC), the Netherlands. Eligibility criteria were adult age (18-50 years), physician-diagnosed asthma, prescription of inhaled corticosteroids ≥3 months in the previous year, access to Internet at home, and the ability to understand written and oral Dutch instructions. Patients who received a maintenance dose of oral corticosteroids were excluded.

All participants were trained in a group educational session to measure lung function as forced expiratory volume in 1 second (FEV_1_) by using a handheld electronic spirometer (PiKo-1, Ferraris Respiratory). After this session, patients were asked to report during a 2-week period FEV_1_ (daily), day and night symptom score (daily), and to fill in at least once weekly an Asthma Control Questionnaire (ACQ) [17] on a specifically designed website or by mobile phone text messaging (SMS). The ACQ is a validated 7-item questionnaire for assessment of actual level of asthma control, consisting of 6 questions on asthma symptoms in the previous 7 days and an FEV_1_ measurement. Optimal cut-point for “well-controlled” is ≤0.75 and a value of ≥1.50 confirms “uncontrolled” asthma [18]. During these 2 weeks, patients did not receive feedback on their actual level of asthma control.

After the 2-week period, all patients were randomized to either IBSM support adjacent to UC, that is, Internet group (IG), or to UC alone. Strategy allocation of patients on a 1:1 ratio was conducted by JKS using a computer-generated permuted block scheme. Patients were stratified on care provider (general practice vs outpatient clinic) and asthma control at baseline. The SMASHING study was powered to detect a difference in the primary outcome asthma-related quality of life, as measured by the Asthma Quality of Life Questionnaire (AQLQ) score [19] between the two groups. Due to the nature of the intervention and its pragmatic character, researchers were not blinded for group allocation. This study was approved by the ethical committee of the LUMC, the Netherlands, and was conducted in concordance with the principles of the Declaration of Helsinki [20], as amended in Seoul 2008. The trial conformed to the Consolidated Standards of Reporting Trials (CONSORT)-eHealth Checklist (Multimedia Appendix 1) [21].

### Internet-Based Self-Management Support Program

The IBSM support program is based on focus groups [22], the Chronic Care model [23], and known key components for effective self-management [5]. The program was aimed at supporting patients in conducting self-management activities and developing a patient-provider partnership in asthma care [3]. Focus groups were conducted to explore barriers for conducting self-management skills and to identify the potential role of an IBSM support tool. In particular, patients with asthma that was not well controlled (ACQ>0.75) were motivated to use novel information and communication technologies for management of their disease. The Chronic Care model is aimed at improving health care outcomes for patients with a chronic disease by means of a proactive patient-professional partnership that addresses both organizational factors (eg, decision support systems) and resources (eg, self-management support). We incorporated modules for electronic monitoring of asthma control and lung function (weekly ACQ and FEV_1_), a personal action plan, communication with a respiratory nurse (RN), and education.

**Figure 1:**
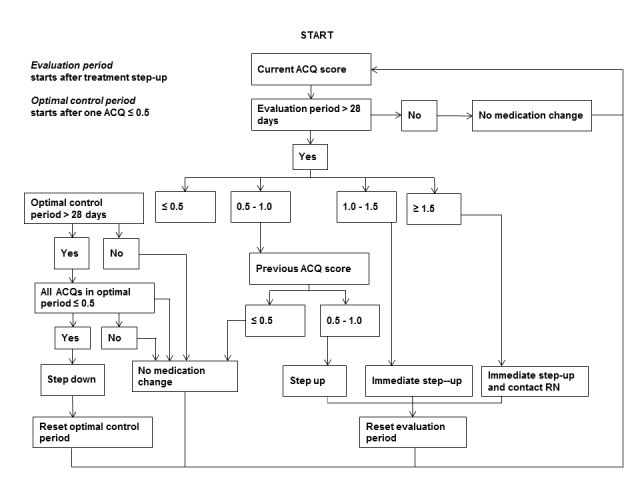
Treatment algorithm.

During 12 months of follow-up, IG patients had access to IBSM support (Multimedia Appendix 2); after this period, IBSM support (including access to the website) was automatically terminated. Patients were instructed on how they could log in by using a personal username and password and how to use their personal action plan. The program included reminder options for monitoring activities (ie, ACQ, day and night symptom score, lung function), which were initially sent once weekly by either email or mobile phone SMS text messaging, but during follow-up frequency could be adjusted according to the preferences of the individual patient. Patients received immediate feedback (to maintain, step up or step down in medication, and/or to contact a health care professional) on self-monitoring outcomes according to a treatment algorithm (Figure 1) and a predefined action plan based on 6 medication steps (Table 1).

**Figure 2:**
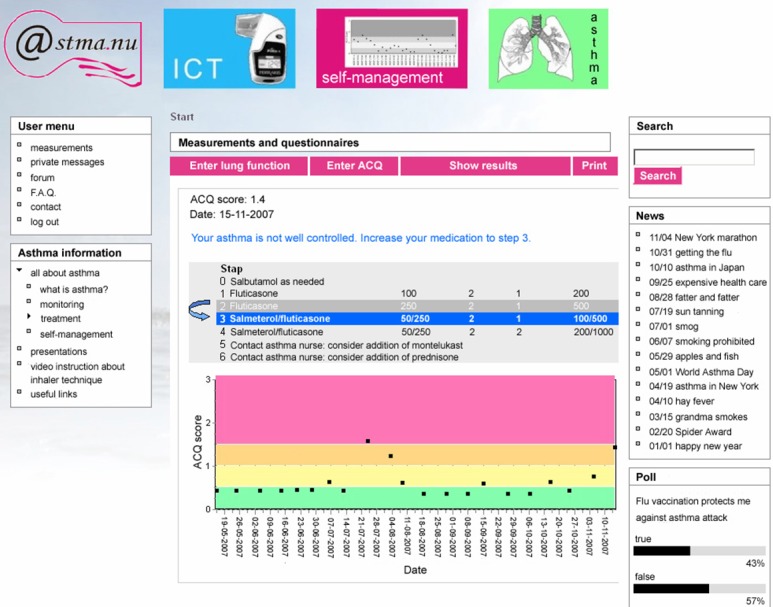
Traffic light display.

Five respiratory physicians, two general practitioners with a particular focus on respiratory diseases, and two respiratory epidemiologists participated in the development of this algorithm. Action plans of patients were based on their actual medication at the time of study enrollment. Treatment steps corresponded with (inter) national guidelines [3,24] on asthma management. Briefly, asthma medication can be aimed at (1) decreasing of airway narrowing (ie, beta2-agonists), and/or (2) decreasing airway inflammation (ie, glucocorticosteroids, leukotriene modifiers). A traffic light display (Figure 2) was used to indicate the level of asthma control: green (well-controlled, ACQ≤0.5), yellow (0.5<ACQ<1.0), orange (1.0<ACQ<1.5), and red (uncontrolled, ACQ score≥1.50).

After each medication change, a 4-week evaluation period commenced during which no advice to change treatment was given, except in the case of symptom deterioration. E-messaging, telephone, or Web-based communication allowed patients to interact with the RN. Additionally, the RN supported patients by nurse-initiated communication characterized by a supportive style to give positive feedback on achieved successes (eg, step-down in medication) or to inquire for reasons on not following treatment advice (eg, side effects). The RN reminded patients to fill in research questionnaires at 12 months of follow-up. On average, the RN spent 1-2 hours per week on patient- and nurse-initiated communication for all IG patients.

Education components of IBSM support were provided both online (eg, educational pages, newsfeed) and offline. Offline education consisted of a group education session that dealt with topics related to asthma self-management, usage of the IBSM tool, and designing an action plan based on current medication. Online information was based on information provided by the Lung Foundation Netherlands. Newsfeed content was kept up to date and contained items related to asthma and management of chronic diseases (eg, healthy lifestyle). During the study, neither major content/functionality changes nor bug fixes were required.

Program content was developed by JKS in close collaboration with the departments of Public Health and Primary Care (LUMC), Respiratory Medicine (LUMC, Amsterdam Medical Center), and Haga Teaching Hospital, The Hague. Software was developed by Furore BV, Amsterdam.

**Table 1:** Medication treatment steps.

Step^a^	Medication
1	As needed rapid-acting beta2-agonist^b^
2	Low-dose inhaled glucocorticosteroids
3a	Low-dose inhaled glucocorticosteroids + long-acting beta2-agonist
3b	Medium-dose inhaled glucocorticosteroids
3c	High-dose inhaled glucocorticosteroids
4a	Medium-dose inhaled glucocorticosteroids + long-acting beta2-agonist
4b	High-dose inhaled glucocorticosteroids + long-acting beta2-agonist
4c	Contact RN or other health care provider: consider addition of leukotriene modifier
5	Contact RN or other health care provider: consider addition of oral glucocorticosteroids

^a^Step numbers correspond with GINA guideline treatment steps [3].

^b^Applies to all treatment steps as this is reliever medication.

### Usual Care

Patients allocated to UC received care as usual by their health care provider. According to the Dutch College of General Practitioners [24], each patient should be provided with a (paper-based) action plan and be invited for a medical review at least once a year.

### Additional Follow-Up 30 Months After Baseline

Patients who previously participated were invited, by a letter containing information on the follow-up measurements, to attend the LUMC for follow-up measurements at 30 months after baseline (Table 2). Nonresponding patients received a reminder letter within 2-4 weeks and an additional telephone call. All participants gave written informed consent during this visit, prior to obtaining measurements. Patients were asked to report on their daily dose of inhaled corticosteroids (ICS) and to complete 2 paper-based questionnaires, namely an ACQ (including FEV_1_) and an AQLQ [19], a validated 21-item questionnaire for assessment of asthma-related quality of life. The minimal clinical important difference for the ACQ is -0.5 and for the AQLQ is 0.5 [25,26]. Both questionnaires have a 7-point scale. Patients were asked to withhold short-acting beta2-agonists for 6-8 hours prior to FEV_1_ measurement. Questionnaires were sent in the mail to patients who were unable or unwilling to attend the LUMC, and an additional home visit was scheduled in case of unavailability of a Piko-1 meter. Inhaled corticosteroid doses were reported as fluticasone equivalents.

### Statistical Analysis

Differences in characteristics at baseline (null months) were analyzed between participants and nonparticipants of both groups (UC and IG) with unpaired *t* tests. ACQ and AQLQ scores, FEV_1_, and daily ICS dose were compared between participants from both groups by applying linear mixed-effect models. ICS doses were reported as fluticasone equivalents. Within- and between-group differences were analyzed with paired and unpaired *t* tests, respectively. Statistical analyses were carried out by intention to treat. For analysis, Stata 9.2 was used. Subgroup analyses were conducted for patients with well-controlled (ACQ≤0.75) and uncontrolled asthma (ACQ>0.75) [17] at baseline.

**Table 2:** Outcome measures.

Characteristics		IBSM support (IG only)			
		Baseline	3 months	12 months	30 months/additional follow-up
**Clinical**					
	Asthma control (ACQ)	X	X	X	X
	Lung function (FEV_1_)	X	X	X	X
	Daily inhaled corticosteroid	X	X	X	X
**Quality of life**					
	Asthma-related quality of life (AQLQ)	X	X	X	X

## Results

### Characteristics

**Figure 3:**
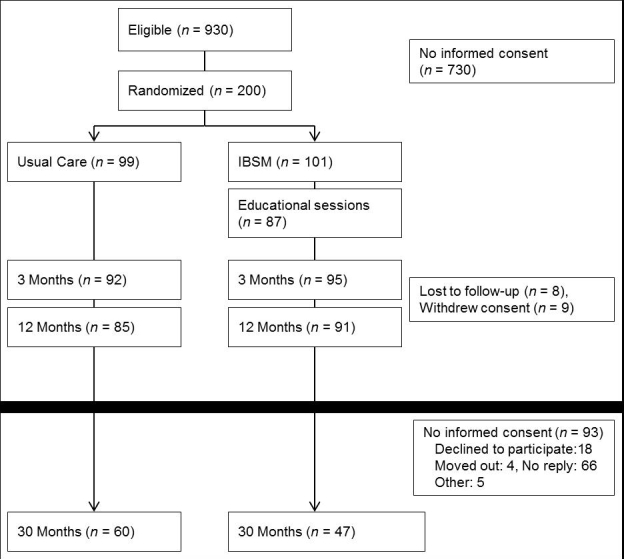
Flowchart of study participants.

In total, 107 out of 200 (54%) invited patients consented to participate for additional follow-up at 1.5 years after finishing the SMASHING study (Figure 3), of whom 60 patients were previously allocated to UC and 47 patients to the IG.

Participants in the IG differed in baseline ACQ scores from nonparticipants (0.93 vs 1.29; *P*=.009). Apart from these differences, no other differences in clinical characteristics at baseline between participants and nonparticipants in each group were identified. Table 2 gives an overview of baseline characteristics of study participants. Baseline characteristics of both groups were similar (Table 3).

### Clinical Outcomes

Twelve months after baseline, significant improved outcomes in favor of the IG were demonstrated for asthma-related quality of life (AQLQ) at 0.37 (95% CI 0.14-0.61) and asthma control of -0.57 (95% CI -0.88 to -0.26) as compared with UC. For those who participated at 30 months, a difference in ACQ score between baseline and 12 months was 0.43 (95% CI 0.21-0.66) in favor of the IG. For those who did not participate at 30 months, a difference between baseline and 12 months of 0.34 (95% CI 0.06-0.61) in favor of the IG was detected. However, there was no significant difference in effect (in ACQ score between 0 and 12 months) between participants (0.43) and nonparticipants (0.33) at 30 months.

**Figure 4:**
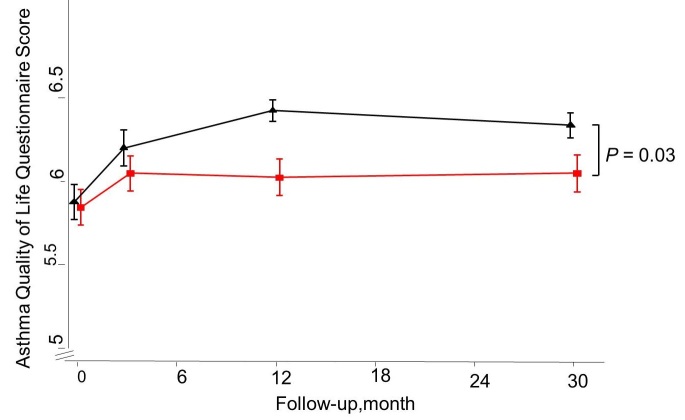
Mean Asthma Quality of Life Questionnaire score for the Internet and Usual care group as measured at 0, 3, 12, and 30 months of follow-up.

**Figure 5:**
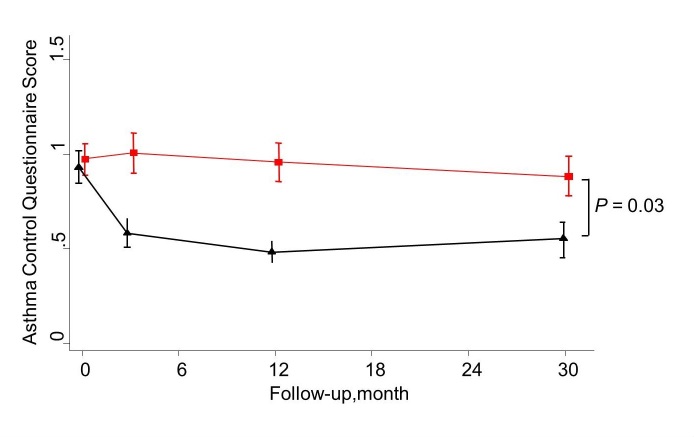
Mean Asthma Control Questionnaire score for the Internet and Usual care group as measured at 0, 3, 12, and 30 months of follow-up.

**Figure 6:**
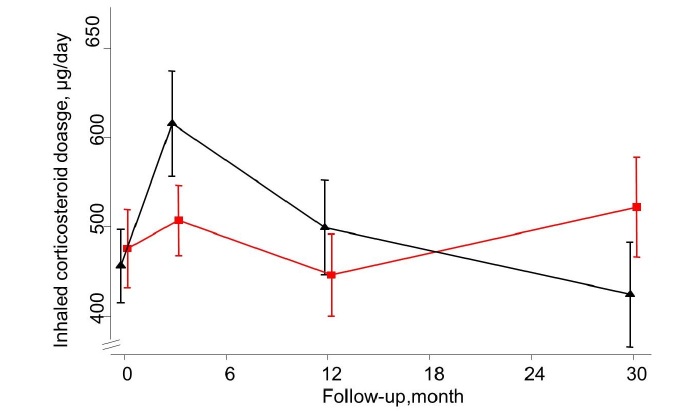
Mean ICS dosage for the Internet and Usual care group as measured at 0, 3, 12, and 30 months of follow-up.

**Figure 7:**
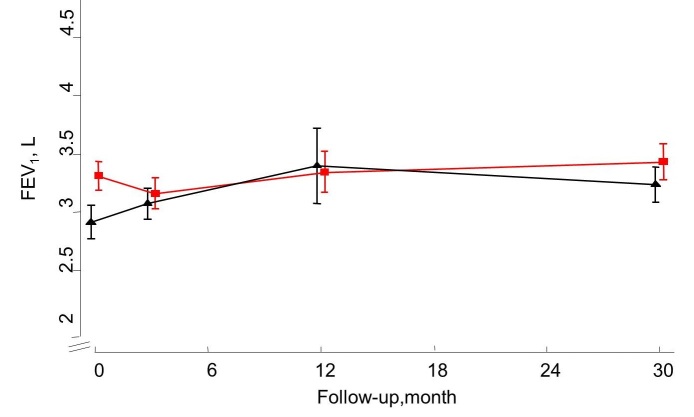
Mean FEV1 for the Internet and Usual care group as measured at 0, 3, 12, and 30 months of follow-up.

At 30 months after baseline, a significant and slightly attenuated improvement was shown for both AQLQ (adjusted between-group difference 0.29 [95% CI 0.01-0.57]) and ACQ (adjusted difference of -0.33 [95% CI -0.61 to -0.05]) scores in favor of the IG (Figures 4 and 5). No such differences were demonstrated for ICS dosage and lung function measured as FEV_1_ (Figures 6 and 7). Patients with uncontrolled asthma at baseline (ACQ≤0.75) had significant better outcomes at 30 months for AQLQ (adjusted within-group difference of 0.52 [95% CI 0.10-0.95]) and asthma control (adjusted difference -0.44 [95% CI 0.04-0.85]) in favor of the IG.

**Table 3:** Baseline characteristics.

Characteristics		Internet group n=47	Usual care group n=60
Age, mean (SD)		36 (8.7)	37 (8.0)
**Gender, n (%)**			
	Male	12 (26)	19 (32)
	Female	35 (74)	41 (68)
Smokers, n (%)		25 (53)	27 (45)
Inhaled corticosteroids (µg/day), mean (SD)		455 (279)	476 (338)
AQLQ score, mean (SD)^a^		5.88 (0.74)	5.84 (0.82)
ACQ score , mean (SD)^b^		0.93 (0.60)	0.97 (0.65)
Prebronchodilator FEV_1_, mean (SD)^c^		3.26 (0.80)	3.41 (0.96)
Prebronchodilator FEV_1_ (% predicted), mean (SD)^c^		96.8 (20)	95.5 (18)

^a^Score range (worst-best), 1-7; minimal clinical important difference (MCID): 0.5.

^b^Score range (worst-best), 6-0; MCID: 0.5.

^c^Number of available observations in the Internet group is 26 and in the usual care group 37.

## Discussion

### Principal Findings

This study indicates that provision of IBSM support for 1 year leads to sustained benefits in terms of asthma control and asthma-related quality of life as compared with usual care, even up to 1.5 years after terminating support. IBSM support was additional to usual care and consisted of education, an action plan, self-monitoring, and regular medical review. Therefore, this study illustrates that sustained health improvements in health can be achieved by a structured care approach and self-management support as outlined by the Chronic Care model [23]. To our knowledge, this is the first study on long-term outcomes of Internet-based comprehensive self-management delivered to patients with asthma in primary care. In the study by Thoonen et al [27], it was demonstrated that guided self-management to patients with asthma in Dutch general practice for 2 years leads to small but sustained benefits in asthma control (except for lung function) and asthma-related quality of life as compared to usual care.

### Limitations

The original SMASHING study had several strengths, which have been discussed at length elsewhere [15]. Briefly, the study had a strong randomized controlled design without major baseline differences between patients in both groups and was characterized by a pragmatic attitude [28]. Nevertheless, results of this follow-up measurement need to be interpreted with some caution. First, our response rate was relatively low compared to other long-term outcome studies [29], which might limit the generalizability of our results. Second, even though a sustained effect for both asthma control and asthma-related quality of life in favor of the IG was demonstrated, these differences did not reach the threshold of a clinical important difference (MCID). However, we included patients with well-controlled asthma at baseline. In contrast, the subgroup of patients with uncontrolled asthma at baseline did show a clinical relevant difference in terms of asthma-related quality of life. Patients with worse asthma control have a larger room for improvement and could therefore be more willing to participate in self-management activities [30].

Even though IG patients had better outcomes in terms of asthma control and asthma-related quality of life as compared with UC patients, this difference was not accompanied with a higher ICS dosage for IG patients. Whether this can be attributed to the ability of the individual IG patient to tailor treatment to his or her need remains unclear. Moreover, the propensity to change in-treatment steps could have been reduced as guidance by IBSM support was terminated.

To what extent demonstrated long-term benefits can be attributed to the mode of delivery, beyond incorporating components (education, monitoring, action plan, and regular review) for effective self-management, remains unclear. More specifically, whether the IBSM approach has led to improved self-management skills can only be postulated as we did not collect data on self-management outcomes, such as self-efficacy or compliance. If self-management skills have been improved, this is due to more intensive support in the start-up phase, since at 12 months after baseline only about 60% of the patients were still using the program on their own initiative [16]. This illustrates that patients do not seem to develop dependence on modern technology, a known barrier for both patients and professionals for using modern technology for asthma self-management support [31]. Moreover, it also suggests that intensity of a self-management support program should be tailored to the individual patient; once a patient has met his or her personal objective, the intensity should be adjusted. Recently, Ryan et al [32] compared a comprehensive self-management approach for adults with poorly controlled asthma (ACQ≥1.5) in general practice in which the monitoring module was either paper-based or mobile phone-based. After 6 months follow-up, both patient groups had improved in terms of asthma control and self-efficacy, but no difference was demonstrated between groups. Clearly, this study does illustrate that there is room for improvement in provision of routine asthma care, since in both groups self-management was delivered in concordance with asthma guidelines (consisting of education, self-monitoring, action plan, and guidance by a professional). Nevertheless, this does not imply that modern technology might not be important since it does offer opportunities to enhance adoption of self-management support within routine care.

### Conclusions

From a patient perspective, there is an increasing demand to use modern technology in the management of chronic disease. In asthma, this is illustrated by a growing number of available apps for asthma “self-management” [33]. Unfortunately, these apps are characterized not only by their lack of all components for effective self-management but also by a lack of reliable content.

Given the sustained benefits, cost-effectiveness becomes more favorable for IBSM, since major intervention costs, such as equipment and education sessions, apply to the first year, while maintenance costs can be spread over a longer period and could be improved by an increased number of users.

For successful adoption of IBSM within routine care and into a patient’s daily life, several preconditions need to be identified and addressed among stakeholders [34,35]. For instance, IBSM support should address daily routines of patients, for example, frequency of monitoring should be able to be adjusted to the needs of the individual patient. From an organizational point of view, an adequate infrastructure for asthma care (eg, routine consultations) should be available within practices. Moreover, technology should be integrated within the current available digital infrastructure. At a professional level, (Internet-based) self-management support requires a proactive role from the health care provider, which allows for a patient-professional partnership. Finally, from an economic point of view, financial incentives such as adequate reimbursements of lung function meters and consultations by health care insurance companies could be considered. More research is needed on the question of how to embed self-management support by means of modern technology within routine practice.

A comprehensive Internet-based self-management approach leads to sustained benefits in terms of asthma-related quality of life and asthma control, even up to 1.5 years after terminating support, particularly for patients with asthma that was not well controlled at baseline. Future research is needed to gain insight on long-term outcomes, cost-effectiveness, and strategies for integration of self-management support by modern technology in real-life settings.

## Multimedia Appendix 1

CONSORT-EHEALTH checklist V1.6.2 [21].

## Multimedia Appendix 2

Screenshots of the original SMASHING website: website functionalities and daily symptom/lung function monitoring; weekly symptom monitoring by using the 7-item Asthma Control Questionnaire; and feedback on symptoms and lung function monitoring.

## Abbreviations

ACQ: asthma control questionnaire
AQLQ: asthma-related quality of life questionnaire
FEV1: forced expiratory volume in one second
GINA: Global Initiative for Asthma
IBSM: Internet-based self-management
ICS: inhaled corticosteroids
IG: Internet group
MCID: minimal clinical important difference
RN: respiratory nurse
SMASHING: Self-Management in Asthma Supported by Hospitals, ICT, Nurses and General practitioners
UC: usual care
