# Supplementary material for: Long-Term Outcomes of Internet-Based Self-Management Support in Adults With Asthma: Randomized Controlled Trial
Source: J Med Internet Res. 2013 Sep 12;15(9):e188. doi: 10.2196/jmir.2640 (PMC3785973; doi:10.2196/jmir.2640)
Supplement: Supplementary file 1 [file jmir_v15i9e188_app1.pdf]

CONSORT-EHEALTH Checklist V1.6.2 Report

(based on CONSORT-EHEALTH V1.6), available at [http://tinyurl.com/consort-ehealth-v1-6].

2640

Date completed

2/19/2013 11:43:39

by

Johanna van Gaalen

Long-term outcomes of providing one-year internet-based self-management support as compared with usual general practice care in adults with asthma. Additional follow-up 1.5 year after a randomized trial

TITLE

1a-i) Identify the mode of delivery in the title

"internet-based"

1a-ii) Non-web-based components or important co-interventions in title

"internet-based self-management support": included weekly asthma control monitoring (online) and treatment advice (online), online and group education and communication (both-on and offline) with a respiratory nurse.

1a-iii) Primary condition or target group in the title

"Adults with asthma"

ABSTRACT

1b-i) Key features/functionalities/components of the intervention and comparator in the METHODS section of the ABSTRACT

Intervention: "One year Internet-based self-management support" (Internet Group):
Comparator: Usual care

1b-ii) Level of human involvement in the METHODS section of the ABSTRACT

"group education and communication (both on- and offline) with a respiratory nurse."

1b-iii) Open vs. closed, web-based (self-assessment) vs. face-to-face assessments in the METHODS section of the ABSTRACT

Recruitment was conducted offline
"200 adults with physician diagnosed asthma (three or more months of inhalation corticosteroids prescribed in the past year) from 37 general practices and one academic outpatient department who previously participated were invited by letter for additional follow-up at 1.5 years after finishing the study."
We used patient reported outcome measurements for assessment of outcomes:
"The Asthma Control Questionnaire (ACQ) and the Asthma Quality of Life Questionnaire (AQLQ) was completed by 107 participants (60 UC participants and 47 IG participants)"

1b-iv) RESULTS section in abstract must contain use data

"200 adults with physician diagnosed asthma (three or more months of inhalation corticosteroids prescribed in the past year) from 37 general practices and one academic outpatient department who previously participated were invited "

"The Asthma Control Questionnaire (ACQ) and the Asthma Quality of Life Questionnaire (AQLQ) was completed by 107 participants (60 UC participants and 47 IG participants)."

1b-v) CONCLUSIONS/DISCUSSION in abstract for negative trials

Not applicable.

INTRODUCTION

2a-i) Problem and the type of system/solution

"Even though effective therapies are widely available many patients do not achieve these treatment goals. A proactive patient-centred approach , consisting of education, treatment goals, self-monitoring, action plan, accompanied with guidance and regular review by a healthcare provider has the potential to improve outcomes in asthma, i.e. improved quality of life, reduced number of hospitalizations and unscheduled doctor visits. In spite of the prominent role within guidelines take-up of this “guided self-management” is lacking. Whilst many practices do offer patients a routine medical review, only a minority of patients are provided with an action plan by their healthcare provider. Usage of action plans by patients could be enhanced if action plans are part of a patient-professional partnership, and when they are tailored to the needs of the individual patient.
Provision of internet-technology has been proposed as an appealing medium for asthma management.

2a-ii) Scientific background, rationale: What is known about the (type of) system

Indeed, in the study by Van der Meer et al. in patients with mild to moderate persistent asthma it was demonstrated that provision of an internet-based self-management (IBSM) support program during one year leads to improved asthma-related quality of life, asthma control, lung function and the number of symptom free days as compared to usual care alone. A post-hoc analysis of this study demonstrated that patients with not well controlled asthma benefited the most from IBSM support. In addition, this study showed that at twelve months of follow-up about 60% of the patients were still using the program on their individual indication. However it is unknown whether the benefits are sustained over a long-term period."

METHODS

3a) CONSORT: Description of trial design (such as parallel, factorial) including allocation ratio

"We hypothesized that benefits of providing one year IBSM support, are sustained over a long-term period.In this article we aim to assess the long-term effects of providing patients one year of IBSM support as compared to usual care alone."

3b) CONSORT: Important changes to methods after trial commencement (such as eligibility criteria), with reasons

Not applicable.

3b-i) Bug fixes, Downtimes, Content Changes

Not applicable for this follow-up measurement. However "neither major content / functionality changes nor bug fixes were required" during the one year of IBSM support.

4a) CONSORT: Eligibility criteria for participants

"Eligibility criteria were: adult age (18-50 years), physician diagnosed asthma, prescription of inhaled corticosteroids ≥ three months in the previous year, access to internet at home and the ability to understand written and oral Dutch instructions. Patients who received a maintenance dose of oral corticosteroids were excluded."

4a-i) Computer / Internet literacy

Other than having "access to internet at home and the ability to understand written and oral Dutch instructions ", we do not have in-depth information on health lliteracy.

4a-ii) Open vs. closed, web-based vs. face-to-face assessments:

Recruitment was conducted offline
"Patients who previously participated were invited by letter, containing information on the follow-up measurements"
Outcomes were assessed by using patient reported outcome measurements:
"Patients were asked to report on their daily dose of inhaled corticosteroids (ICS) and to complete two paper-based questionnaires, namely an ACQ (including FEV1) and an asthma quality of life questionnaire (AQLQ), a validated 21-item questionnaire for assessment of asthma related quality of life. Both questionnaires have a seven point scale. Patients were asked to withhold short-acting β2-agonists 6-8 hours prior to FEV1 measurement. Postal questionnaires were sent to patients who were unable or unwilling to attend the LUMC and an additional home visit was scheduled in case of unavailability of a Piko-1 meter."

4a-iii) Information giving during recruitment

"Patients who previously participated were invited by letter, containing information on the follow-up measurements, to attend the Leiden University Medical Center (LUMC) for follow-up measurements. Non responding patients received a reminder letter within two to four weeks and an additional telephone call. All participants gave written informed consent during this visit, prior to obtaining measurements."

4b) CONSORT: Settings and locations where the data were collected

Either in the LUMC or at home:
"patients who previously participated were invited by letter, containing information on the follow-up measurements, to attend the LUMC for follow-up measurements."
"Postal questionnaires were sent to patients who were unable or unwilling to attend the LUMC and an additional home visit was scheduled in case of unavailability of a Piko-1 meter."

4b-i) Report if outcomes were (self-)assessed through online questionnaires

Outcomes were assessed by using patient reported outcome measurements (see item 4a-ii).

4b-ii) Report how institutional affiliations are displayed

Patients received an information letter, signed by the research team and containing the letterhead of the LUMC.

5) CONSORT: Describe the interventions for each group with sufficient details to allow replication, including how and when they were actually administered

5-i) Mention names, credential, affiliations of the developers, sponsors, and owners

"Program content has been developed by JKS in close collaboration with departments of Public health and primary care (LUMC), Respiratory medicine (LUMC, Amsterdam Medical Center (AMC)) and Haga Teaching Hospital, The Hague, the Netherlands. Software has been developed by Furore BV, Amsterdam, the Netherlands."
"This study is supported by grants from the Netherlands Organization for Health Research and Development (ZON-MW) and the Lung Foundation Netherlands."

5-ii) Describe the history/development process

"The IBSM support program is based on focus groups, the Chronic Care model and known key components for effective self-management. The program was aimed at supporting patients in conducting self-management activities and to develop a patient-provider partnership in asthma care. Focus groups were conducted to explore barriers for conducting self-management skills and to identify the potential role of an IBSM support tool. Particularly patients with not well controlled asthma (ACQ >0.75) were motivated to use novel information and communication technologies for management of their disease. The Chronic Care model is aimed at improving healthcare outcomes for patients with a chronic disease by means of a proactive patient-professional partnership by addressing both organizational factors (i.e. decision support systems) and resources (i.e. self-management support). We incorporated modules for electronic monitoring of asthma control and lung-function (weekly ACQ and FEV1), a personal action plan, communication with a respiratory nurse (RN) and education."

5-iii) Revisions and updating

Not applicable for this follow-up measurement.

5-iv) Quality assurance methods

Program content:
"Program content has been developed by JKS in close collaboration with departments of Public health and primary care (LUMC), Respiratory medicine (LUMC, Amsterdam Medical Center (AMC)) and Haga Teaching Hospital, The Hague, the Netherlands."

Assessment of asthma control:
"The ACQ is a validated seven item questionnaire for assessment of actual level of asthma control, consisting of six questions on asthma symptoms in the previous seven days and a FEV1 measurement."

Treatment alogrithm & treatment plan:
"Five respiratory physicians, two general practitioners with a particular focus on respiratory diseases and two respiratory epidemiologists participated in the development of this algorithm. Action plans of patients were based on their actual medication at time of study enrollment. Treatment steps corresponded with (inter-) national guidelines on asthma management."

Information:
"Online information was based on information provided by the Lung Foundation Netherlands."

5-v) Ensure replicability by publishing the source code, and/or providing screenshots/screen-capture video, and/or providing flowcharts of the algorithms used

Not applicable, the website went offline in 2007.
See Multimedia appendix 1 for screenshots.

5-vi) Digital preservation

Not applicable, the website went offline in 2007.
See Multimedia appendix 1 for screenshots.

5-vii) Access

"Patients could log in by using a personal username and password."

5-viii) Mode of delivery, features/functionalities/components of the intervention and comparator, and the theoretical framework

Theoretical framework:
"The IBSM support program is based on focus groups, the Chronic Care model and known key components for effective self-management."

Tailoring to the individual patient's need:
By means of the treatment algorithm and predefined medication plan

.

Feedback

Asthma control:
"A traffic light display was used to indicate the level of asthma control: green (well controlled, ACQ ≤ 0.5), yellow (0.5 < ACQ < 1.0), orange (1.0 < ACQ < 1.5) and red (poorly controlled, ACQ score ≥ 1.50)."

Treatment advice:
"Patients received immediate feedback (either to maintain, step-up and/or to contact a healthcare professional or step-down in medication) on self-monitoring outcomes according to a treatment algorithm (Figure 1) and a predefined action plan based on six medication steps (Table 1)."

Communication:
"E-messaging, telephone or web based communication allowed patients to interact with the RN. Additionally the RN supported patients by nurse initiated communicated characterized by a supportive style to give positive feedback on achieved successes (i.e. step-down in mediation) or to inquire for reasons on not following treatment advice (i.e. side effects)."

5-ix) Describe use parameters
Not applicable for this follow-up measurement.

During the one year of IBSM support patients were initially adviced to monitor their level of asthma control regurlarly, in order to get acquainted with conducting monitoring activities and to gain insight in their level of asthma control and optimal lung function.

"A post-hoc analysis of this study [SMASHING study] demonstrated that patients with not well controlled asthma benefited the most from IBSM support. In addition, this study showed that at twelve months of follow-up about 60% of the patients were still using the program on their individual indication."

5-x) Clarify the level of human involvement
Baseline (both groups)
"All participants were trained in a group educational session to measure lung function as forced expiratory volume in one second (FEV1) by using a hand held electronic spirometer (PiKo-1, Ferraris Respiratory, Hertford, United Kingdom)."

"In the UC group patients received care as usual. According to The Dutch College of General Practitioners advices to medical review patients at least once a year and frequency should be increased if asthma is not well uncontrolled treatment goals have been achieved; and it advices to provide patients with a written action plan."

Internet group only:
"Patients were instructed on how they could log in by using a personal username and password and how to use their personal action plan."
"E-messaging, telephone or web based communication allowed patients to interact with the RN. Additionally the RN supported patients by nurse initiated communicated characterized by a supportive style to give positive feedback on achieved successes (i.e. step-down in mediation) or to inquire for reasons on not following treatment advice (i.e. side effects)."
"On average the RN spent one to two hours a week on patient- and nurse initiated communication for all IG patients."

5-xi) Report any prompts/reminders used
"The RN reminded patients to fill in research questionnaires at twelve months of follow-up."

"The program included options for reminders on monitoring activities (ACQ, day-and night symptom score, lung function), which were initially sent once weekly by either e-mail or mobile phone text messaging but during follow-up frequency could be adjusted according to the preferences of the individual patient."

5-xii) Describe any co-interventions (incl. training/support)
See item 5-x.

6a) CONSORT: Completely defined pre-specified primary and secondary outcome measures, including how and when they were assessed

"Patients were asked to report on their daily dose of inhaled corticosteroids (ICS) and to complete two paper-based questionnaires, namely an ACQ (including FEV1) and an asthma quality of life questionnaire (AQLQ) [17], a validated 21-item questionnaire for assessment of asthma related quality of life. The minimal clinical important difference for the ACQ is – 0.5 and for the AQLQ 0.5and AQLQ [21, 22]. Both questionnaires have a seven point scale. Patients were asked to withhold short-acting β2-agonists 6-8 hours prior to FEV1 measurement. Postal questionnaires were sent to patients who were unable or unwilling to attend the LUMC and an additional home visit was scheduled in case of unavailability of a Piko-1 meter."

SMASHING study:
"The SMASHING study was powered to detect a difference in the primary outcome asthma-related quality of life as measured by the Asthma Quality of Life Questionnaire (AQLQ) score between the two groups." Secondary (clinical) outcomes included:asthma control, sympt-m-free days, pre-bronchodilator FEV1, daily inhaled corticosteroid dose and exacerbations.

6a-i) Online questionnaires: describe if they were validated for online use and apply CHERRIES items to describe how the questionnaires were designed/deployed

" Patients were asked o complete two paper-based questionnaires, namely an ACQ [..] asthma quality of life questionnaire (AQLQ), a validated 21-item questionnaire for assessment of asthma related quality of life"

"The ACQ is a validated seven item questionnaire for assessment of actual level of asthma control, consisting of six questions on asthma symptoms in the previous seven days and a FEV1 measurement. Optimal cut-point for 'controlled' is equals or less than 0.75 and a value of ≥1.50 confirms 'uncontrolled' asthma."

6a-ii) Describe whether and how “use” (including intensity of use/dosage) was defined/measured/monitored

Not applicable for this follow-up measurement.

6a-iii) Describe whether, how, and when qualitative feedback from participants was obtained

Not applicable for this follow-up measurement.

6b) CONSORT: Any changes to trial outcomes after the trial commenced, with reasons

Not applicable. "Full details of the study methodology and subjects for the Self-Management of Asthma Supported by Hospitals, ICT, Nurses and General Practitioners (SMASHING) study have been published elsewhere*"

*Van der Meer V, Bakker MJ, van den Hout WB, Rabe KF, Sterk PJ, Kievit J, Assendelft WJ, Sont JK, Group SS: Internet-based self-management plus education compared with usual care in asthma: a randomized trial. Ann Intern Med 2009, 151:110-120.

7a) CONSORT: How sample size was determined

7a-i) Describe whether and how expected attrition was taken into account when calculating the sample size

Not applicable for this follow-up measurement.

7b) CONSORT: When applicable, explanation of any interim analyses and stopping guidelines

Not applicable for this follow-up measurement.

8a) CONSORT: Method used to generate the random allocation sequence

Not applicable for this follow-up measurement.

"Full details of the study methodology and subjects for the Self-Management of Asthma Supported by Hospitals, ICT, Nurses and General Practitioners (SMASHING) study have been published elsewhere. [.] Strategy allocation of patients on a 1:1 ratio was conducted by JKS by using a computer-generated, permuted block-scheme. Patients were stratified on care provider (general practice vs. outpatient clinic) and asthma control at baseline."

8b) CONSORT: Type of randomisation; details of any restriction (such as blocking and block size)

Not applicable for this follow-up measurement (see item 8a).

9) CONSORT: Mechanism used to implement the random allocation sequence (such as sequentially numbered containers), describing any steps taken to conceal the sequence until interventions were assigned

Not applicable (see item 8a)

10) CONSORT: Who generated the random allocation sequence, who enrolled participants, and who assigned participants to interventions

Random allocation sequence generation and strategy allocation: not applicable (see item 8a).

WR and PR enrolled participants for this follow-up measurement.

11a) CONSORT: Blinding - If done, who was blinded after assignment to interventions (for example, participants, care providers, those assessing outcomes) and how

11a-i) Specify who was blinded, and who wasn’t

"Due to the nature of the intervention and its pragmatic character, researchers were not blinded for group allocation. "

11a-ii) Discuss e.g., whether participants knew which intervention was the “intervention of interest” and which one was the “comparator”

At the time of this follow-up measurement participants were aware of their previous group allocation.

For recruitment of the SMASHING study patients were aware of the content of both strategies as this was clearified in the invitation letter.

11b) CONSORT: If relevant, description of the similarity of interventions

"During twelve months follow-up IG patients had access to IBSM support (Multimedia appendix 1), after this period IBSM support was automatically terminated." During the 18 months after this period, prior to the follow-up measurement, patients in both groups had received care as usual.

12a) CONSORT: Statistical methods used to compare groups for primary and secondary outcomes

"ACQ and AQLQ scores, FEV1, daily ICS dose were compared between participants from both groups by applying linear mixed-effect models. ICS doses were reported as fluticasone equivalents. Within- and between-group differences were analysed with paired and unpaired t tests, respectively. For analysis Stata 9.2 (StataCorp, College Station TX, USA) was used."

12a-i) Imputation techniques to deal with attrition / missing values

Not applicable.

12b) CONSORT: Methods for additional analyses, such as subgroup analyses and adjusted analyses

"Subgroup analyses were conducted for patients with well controlled (ACQ equals or less than 0.75) and not well controlled asthma (ACQ > 0.75) [15] at baseline."

RESULTS

13a) CONSORT: For each group, the numbers of participants who were randomly assigned, received intended treatment, and were analysed for the primary outcome

In total 107 out of 200 (54%) invited patients consented to participate for additional follow-up at 1.5 years after finishing the SMASHING study (figure 2), of whom 60 persons were previously allocated to UC and 47 persons to the IG.

13b) CONSORT: For each group, losses and exclusions after randomisation, together with reasons

No informed consent (n = 93); Declined to participate (n = 3); Moved out (n = 4); No reply (n = 66); Other (n = 5).

13b-i) Attrition diagram

Not applicable.

14a) CONSORT: Dates defining the periods of recruitment and follow-up

Patients were in the beginning of 2008 invited for follow-up at 1.5 year after terminating IBSM support (March 2008).

14a-i) Indicate if critical “secular events” fell into the study period

Not applicable.

14b) CONSORT: Why the trial ended or was stopped (early)
Not applicable.
15) CONSORT: A table showing baseline demographic and clinical characteristics for each group
See table 1.
15-i) Report demographics associated with digital divide issues
See item 4a-i.
Other than having "access to internet at home and the ability to understand written and oral Dutch instructions ", we do not have in-depth information on health lliteracy.
16a) CONSORT: For each group, number of participants (denominator) included in each analysis and whether the analysis was by original assigned groups
16-i) Report multiple "denominators" and provide definitions
See figure 2.
"In total 107 out of 200 (54%) invited patients consented to participate for additional follow-up at 1.5 years after finishing the SMASHING study (figure 2), of whom 60 persons were previously allocated to UC and 47 persons to the IG."
Analysis was by orginal assigned groups.
16-ii) Primary analysis should be intent-to-treat
Not applicable for this follow-up measurement.
17a) CONSORT: For each primary and secondary outcome, results for each group, and the estimated effect size and its precision (such as 95% confidence interval)
"At 30 months after baseline a significant and slightly attenuated improvement was shown for both AQLQ (adjusted between-group difference 0.29 [95% CI 0.01 to 0.57] and ACQ (adjusted difference of -0.33 [95% CI -0.61 to -0.05] scores in favor of the IG. No such differences were demonstrated for ICS dosage and lung function as measured as FEV1 (figure 2).
17a-i) Presentation of process outcomes such as metrics of use and intensity of use
Not applicable.
17b) CONSORT: For binary outcomes, presentation of both absolute and relative effect sizes is recommended
Not applicable.
18) CONSORT: Results of any other analyses performed, including subgroup analyses and adjusted analyses, distinguishing pre-specified from exploratory
"Patients with not well controlled asthma at baseline (ACQ <0.75) had significant better outcomes at 30 months for asthma related quality of life (adjusted within group difference of 0.52 [95% CI 0.10 to 0.95] and asthma control (adjusted difference -0.44 [95% CI 0.04 to 0.85]) in favor of the IG."
18-i) Subgroup analysis of comparing only users
"Patients with not well controlled asthma at baseline (ACQ equals or less than 0.75)"
19) CONSORT: All important harms or unintended effects in each group
Not applicable.
19-i) Include privacy breaches, technical problems
Not applicable.
19-ii) Include qualitative feedback from participants or observations from staff/researchers
Not applicable.

DISCUSSION

20) CONSORT: Trial limitations, addressing sources of potential bias, imprecision, multiplicity of analyses
20-i) Typical limitations in ehealth trials
"Firstly, our sample size was small [..]which makes it difficult to generalize our data.
"Secondly, even though a sustained effect for both asthma control and asthma related quality of life in favor of the IG was demonstrated, these differences did not reach the threshold of a clinical important difference (MCID)."
21) CONSORT: Generalisability (external validity, applicability) of the trial findings
21-i) Generalizability to other populations
"our sample size was small [..] which makes it difficult to generalize our data."
"limited data on health literacy"
"Despite these pitfalls, our finding that patients with initially not well controlled asthma at baseline benefited the most as compared with usual care in terms of asthma related quality of life and asthma control is not surprising. Patients with worse asthma control have a larger room for improvement might, and could therefore be more willing to participate in self-management activities."

However an increasing number of patients is using modern technology in their daily life:
"Modern technology could offer opportunities to enhance take-up of self-management support within routine practice. Additionally, from a patient perspective there is an increasing demand to use modern technology in the management of their chronic disease."
21-ii) Discuss if there were elements in the RCT that would be different in a routine application setting
Not applicable for this follow-up measurement. However, several aspects need to be addressed in order to integrate IBSM support within usual care:
"from an organizational point of view an adequate infrastructure for asthma care (i.e. routine consultations) should be available within practices. Moreover, technology should be integrated within the current available digital infrastructure."
22) CONSORT: Interpretation consistent with results, balancing benefits and harms, and considering other relevant evidence
22-i) Restate study questions and summarize the answers suggested by the data, starting with primary outcomes and process outcomes (use)
"This study indicates that provision of one year IBSM support leads to sustained benefits in terms of asthma control and asthma-related quality of life as compared with usual care, even up to 1.5 year after terminating support. Internet-based self-management was adjacent to usual general practice care and consisted of education, an action plan, self-monitoring and regular medical review"
22-ii) Highlight unanswered new questions, suggest future research
"For a successful take-up of IBSM within routine care and into a patient's daily life, several preconditions need to be identified and addressed amongst stakeholders."
"Future research is needed to gain insight on long-term outcomes, cost-effectiveness and on strategies for integration of self-management support delivered by modern technology in real life settings.

Other information

23) CONSORT: Registration number and name of trial registry
Trial registration: ISCRTN79864465

http://www.controlled-trials.com

24) CONSORT: Where the full trial protocol can be accessed, if available
A summary of the study protocol can be found at:http://www.controlled-trials.com.

25) CONSORT: Sources of funding and other support (such as supply of drugs), role of funders
"This study is supported by grants from the Netherlands Organization for Health Research and Development (ZON-MW) and the Lung Foundation Netherlands. Funding for this publication was obtained from the Netherlands Organization for Scientific Research (NWO) Incentive fund Open Access publications."

X26-i) Comment on ethics committee approval
"This study was approved by the ethical committee of the LUMC, the Netherlands and was conducted in concordance with the principles of the Declaration of Helsinki, as has been amended in Seoul 2008."

x26-ii) Outline informed consent procedures
"Patients who previously participated were invited by letter, containing information on the follow-up measurements, to attend the LUMC for follow-up measurements. Non responding patients received a reminder letter within two to four weeks and an additional telephone call. All participants gave written informed consent during this visit, prior to obtaining measurements."

X26-iii) Safety and security procedures
Not applicable.

X27-i) State the relation of the study team towards the system being evaluated
"JKS was responsible for development of program content, but has no commercial interest"
