# Supplementary figures and images for: Long-Term Outcomes of Internet-Based Self-Management Support in Adults With Asthma: Randomized Controlled Trial
Source: J Med Internet Res. 2013 Sep 12;15(9):e188. doi: 10.2196/jmir.2640 (PMC3785973; doi:10.2196/jmir.2640)

## Slide 1
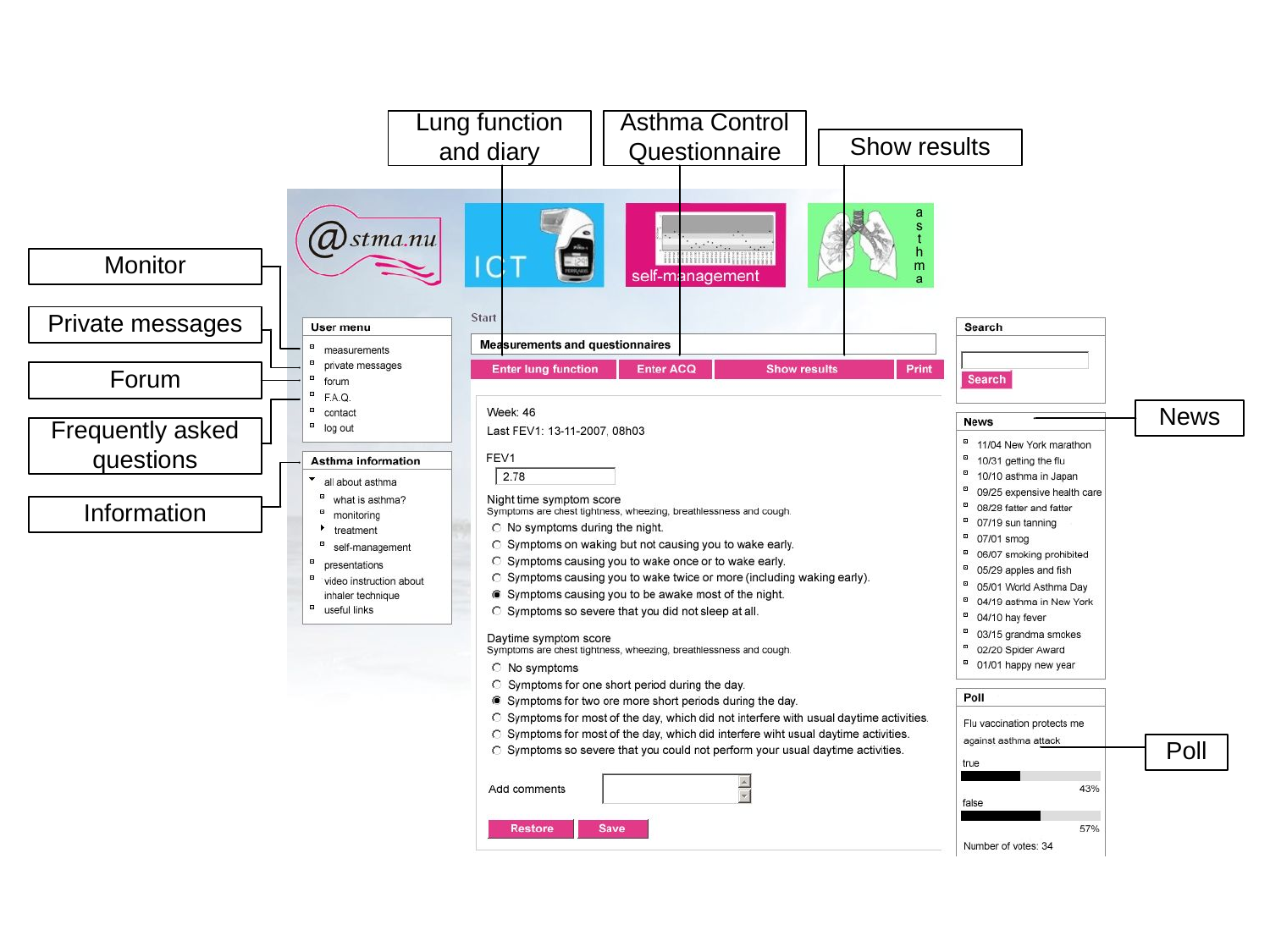

## Slide 2
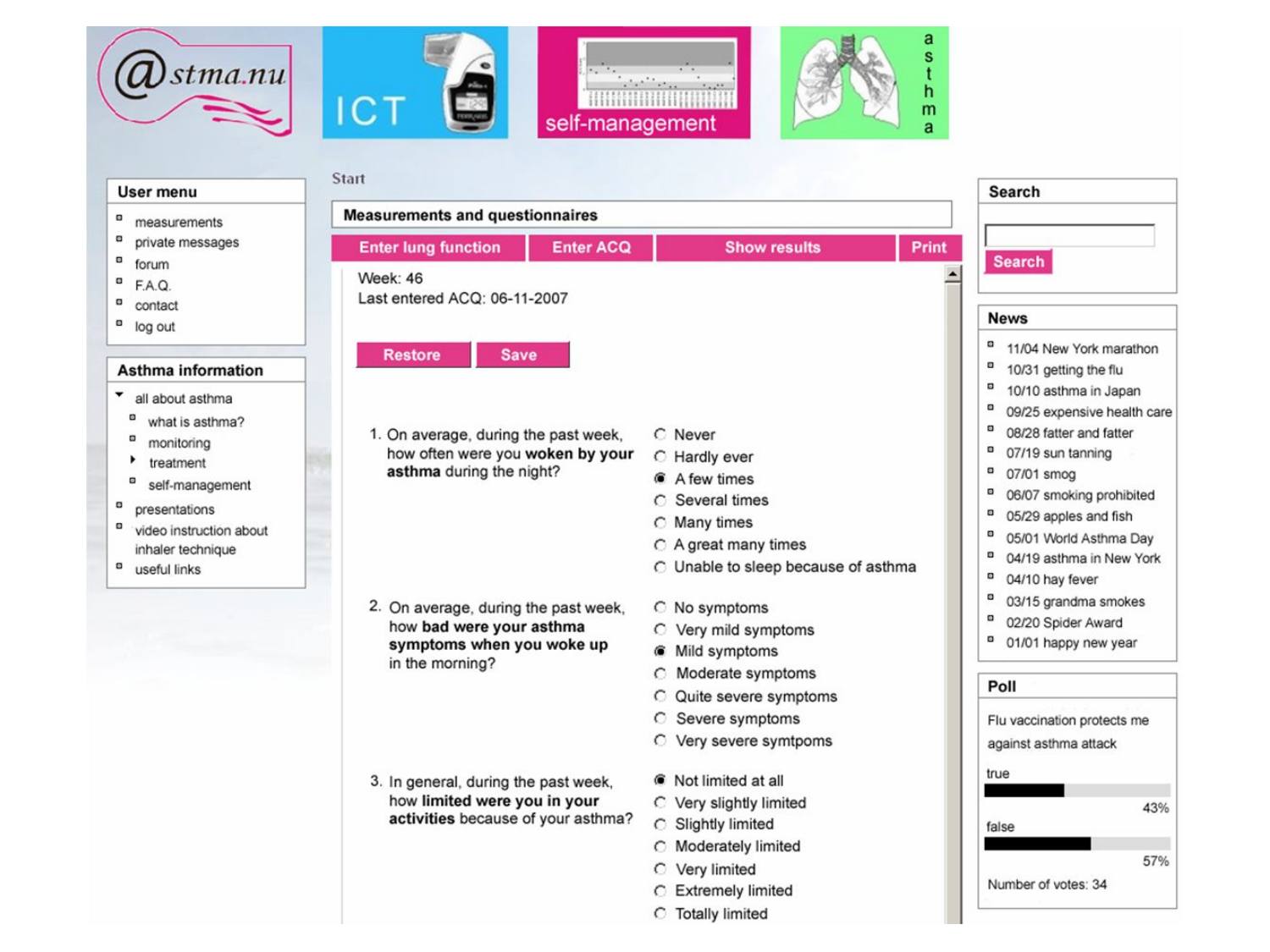

## Slide 3
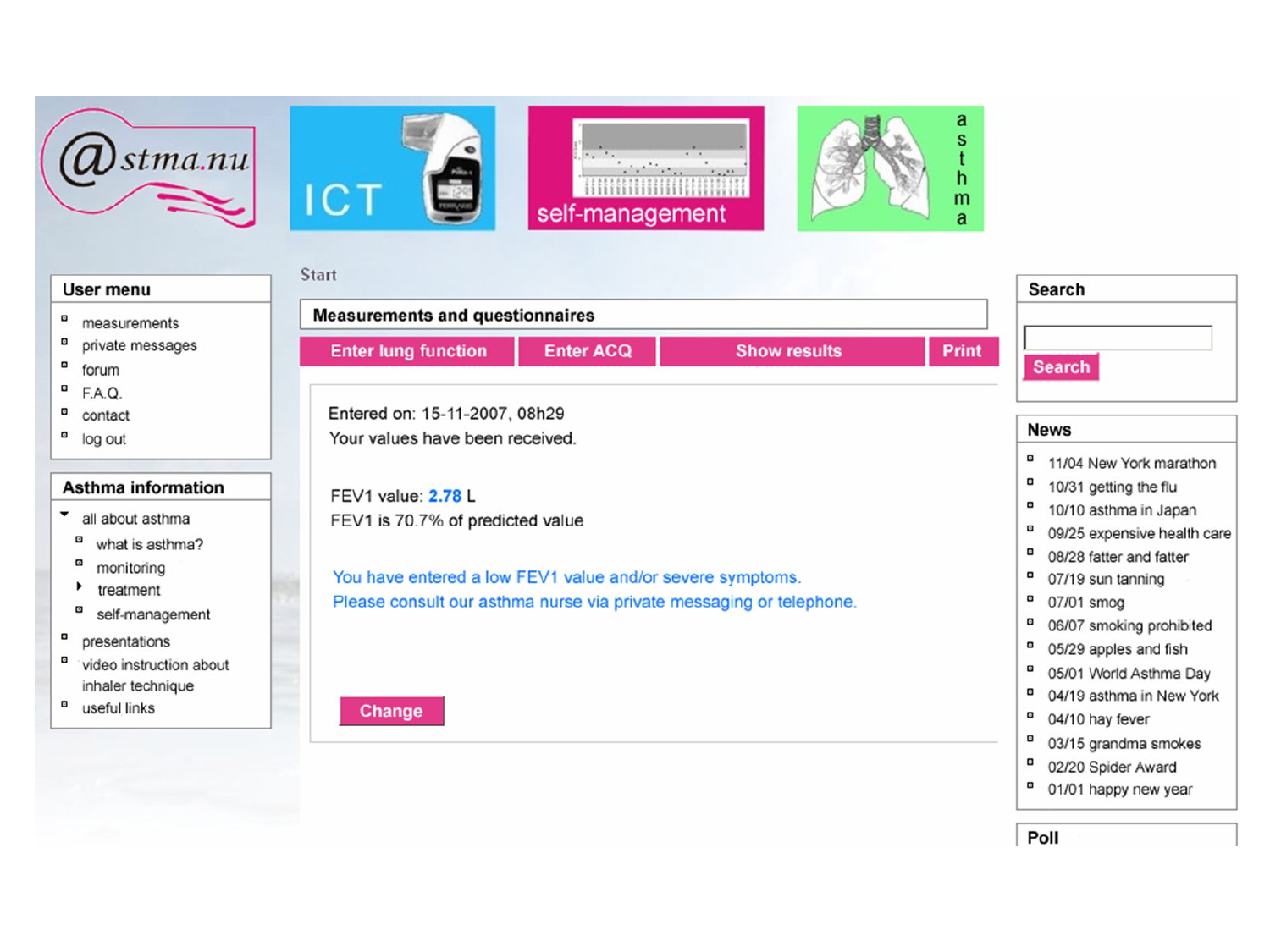

Supplement: Supplementary file 2 [file jmir_v15i9e188_app2.pptx]
